# Expression and Characterization of Recombinant Ecarin

**DOI:** 10.1007/s10930-012-9409-6

**Published:** 2012-04-17

**Authors:** Anna Jonebring, Ute Lange, Elke Bucha, Johanna Deinum, Margareta Elg, Ann Lövgren

**Affiliations:** 1 AstraZeneca R&D, 43183 Mölndal, Sweden; 2 JenAffin GmbH, Winzerlaer Straße 2, 07745 Jena, Germany

**Keywords:** Recombinant ecarin, Prothrombin activator, Kinetic parameters, Protease, Pro-protein activation

## Abstract

The snake venom protease ecarin from *Echis carinatus* was expressed in stable transfected CHO-S cells grown in animal component free cell culture medium. Recombinant ecarin (r-ecarin) was secreted from the suspension adapted Chinese Hamster Ovary (CHO-S) host cells as a pro-protein and activation to the mature form of r-ecarin occurred spontaneously during continued incubation of the cell culture at 37 °C after death of the host cells. Maximal ecarin activity was reached 7 days or more after cell culture viability had dropped to zero. The best producing CHO-S clone obtained produced up to 7,000 EU ecarin/litre in lab scale shaker cultures. The conversion of different concentrations of both prothrombin and prethrombin-2 as substrates for native and r-ecarin were examined with a chromogenic thrombin substrate. At low concentrations both these proteins were converted into thrombin by the two ecarin preparations with comparable rates. However, with prothrombin concentrations above 250 nM r-ecarin apparently had a two times higher turnover than native ecarin, consistent with the observed rapid complete conversion of prothrombin into thrombin by r-ecarin. With r-ecarin a *K*_m_ value of 0.4 μM prethrombin-2 was determined but only a rough estimate could be made of the *K*_m_ for prothrombin of 0.9 μM. In conclusion, r-ecarin was identified as a promising candidate for replacement of native ecarin in assays utilizing conversion of prothrombin to thrombin.

## Introduction

There is a clinical need to be able to quantify both functional and non-functional prothrombin in plasma. Functionally active prothrombin contains a GLA-domain containing ten glutamic acid residues that are post-translationally converted into γ-carboxyglutamate (Glx). Inactive prothrombin that lacks Glx residues is in vivo present under conditions of cancer [2, 3, 11] or by the use of warfarin [10]. In vivo, thrombin is generated only from functionally active prothrombin in series of cleavage steps by coagulation factor Xa (FXa) cleavage in a GLA-domain and Ca^2+^-dependent process, giving rise to different intermediates as prethrombin-2 or meizothrombin.

Ecarin is a component of the venom from the saw-scaled viper *Echis carinatus* that can convert prethrombin-2 to thrombin [12] or prothrombin to meizothrombin, which subsequently will be converted to thrombin by auto-catalytic activity [8]. In contrast to FXa-like snake venom prothrombin activators, the ecarin protease activity does not depend on additional co-factors or calcium ions neither on the presence of a GLA domain [9]. Therefore, ecarin purified from snake venom is currently used in vitro as prothrombin activator in diagnostic reagents for measurement of total prothrombin in plasma or buffer [1] and for quantitative determination of direct thrombin inhibitors [5].

The cloning of the ecarin mRNA sequence was published more than 15 years ago [6] and recombinant GLA-domain–less prethrombin-2 digested with recombinant ecarin has been used to produce recombinant human thrombin [12]. However, to our knowledge, very little work has been presented on the characterization of recombinant ecarin (r-ecarin), and, nothing has previously been published on how different prothrombin fragments and prothrombin itself compare as substrates for r-ecarin, compared to venom-prepared native ecarin. Here we show that r-ecarin should be a better alternative for diagnostic use than the currently used venom prepared enzyme.

## Materials and Methods

### Materials

#### Proteins

Purified human prothrombin was from JenAffin GmbH (Jena, Germany) or, from Enzyme Research Laboratories (South Bend, IN, USA) and human prethrombin-2 protein was obtained from Abcam PLC (Cambridge, UK), Prod. No. ab62535. The proteins were stored at −20 °C and rapidly thawed at 37 °C. Protein solutions containing different concentrations were prepared by dilution in Tris buffer consisting of 0.05 M Tris HCl, 0.1 M NaCl, pH 8 at 37 °C. The protein concentration after buffer exchange and dilution of stock solutions was determined from the absorbance spectrum between 250 and 350 nm, according to the E_280_ provided by the supplier. Native ecarin, prepared from the venom of *E*. *carinatus,* was from DSM Nutritional Products Ltd. Branch Pentapharm (Basel, Switzerland) and from Sigma Aldrich (St Louis, USA). Ecarin was dissolved in 0.154 M NaCl to 10 EU/ml and stored in aliquots at −20 °C. Recombinant ecarin was the crude cell culture supernatants from the cell line 11B9b. Fresh ecarin solutions were prepared on the day of the experiments (0.1 EU ecarin/ml).

#### Chemicals

The chromogenic thrombin substrate H-CHG-Ala-Arg-pNa, was from JenAffin GmbH and stored in Tris buffer at +2 to +8 °C. The second chromogenic thrombin substrate S-2238 (H-D-Phe-Pip-Arg-pNa^.^2HCl) was purchased from Aniara Corp. (Ohio, USA). All chemicals were reagent grade.

### Methods

#### Thrombin Activity Measurements

By hydrolysis of the chromogenic thrombin substrates H-CHG-Ala-Arg-pNa and S-2238 free pNA (paranitroaniline) is generated that is monitored by the change in absorbance at 405 nm (A_405_) in time (t) by spectrophotometry under conditions that the rate, dA405/dt, is linear in the concentration of thrombin.

#### Production of r-Ecarin in CHO Cells

An ecarin encoding sequence optimized for expression in mammalian cells was synthesized and cloned into the Invitrogen vector pCDNA 3.1+. The complete expression vector sequence is available under data base accession FW582517.1. The translated amino acid sequence from the optimized nucleotide sequence was the same as the one reported by Nishida et al. [6]. This construct was used to stably transfect CHO-S cells, obtained from Invitrogen, according to procedures recommended by the cloning vector supplier (Invitrogen, Life Technologies, UK). The culture medium was CD-CHO from Invitrogen, and was supplemented with Glutamax, HT-supplement and non-essential amino acids as recommended by Invitrogen. The cells were grown at 37 °C in an atmosphere containing 5 % carbon dioxide.

Clones were generated by limiting dilution cloning. To screen for r-ecarin producing clones small samples from the culture supernatant were removed and mixed with 10 mg/l prothrombin to a final concentration of 1 mg/l prothrombin in assay buffer (50 mM Tris–HCl, pH 7.4 containing 0.1 % BSA) and incubated 20–40 min at 37 °C. The generated thrombin was then detected by addition of an equal volume of 1–2 mM solution of S-2238 in a 96 well Nunc F plate at room temperature. The increase in absorbance at 405 nm was monitored and the reaction was stopped when suitable by addition of acetic acid (5 % final concentration). To produce r-ecarin for characterization, clone 11B9b was grown in culture medium in shake flasks until the cells were no longer viable (approximately 7 days). After cell death, incubation at 37 °C was continued for at least 7 more days. The final activity of the produced r-ecarin was quantified in the assay buffer at pH 7.4 and at 37 °C against a standard of venom derived ecarin from Sigma. The activity of r-ecarin was compared with the JenAffin internal native ecarin standard in Tris buffer at pH 8.

#### Western Blot Detection of Ecarin

Strep-tagged mature ecarin (aa 191-616) was produced in *Escherichia coli* by cloning the mature ecarin sequence into the expression vector pASK-IBA2 and purifying the ecarin essentially as described by the vector supplier (IBA GmbH, Germany). The purified r-ecarin was used for raising polyclonal antisera in rabbits. For western blots cell culture samples were reduced and run on 4–12 % SDS-PAGE gradient gels and blotted onto nitrocellulose membranes. Membranes were blocked with 5 % BSA in PBS buffer and labelled with rabbit anti-ecarin serum and an anti-rabbit immunoglobulin ALP-conjugate (Sigma cat. no. A2306). Staining was done with BCIP/NBT (Sigma cat. no. B5655).

#### Ecarin Activity Determination

For the determination of the ecarin activity the activation of human prothrombin was evaluated in a simplified spectrophotometric assay, using a chromogenic thrombin substrate at 37 °C. Since the generated activation products from prothrombin, meizothrombin and thrombin, can cleave H-CHG-Ala-Arg-pNa with similar activity [4] the ecarin activity is measured from the change in A_405_ in time. For estimation of the ecarin units and for the comparison of the properties of r-ecarin and native ecarin the activity was determined by measuring the rate from the time until a threshold of absorbance, A_405_ = 0.1, was reached.

#### Ecarin Enzyme Kinetics

The characterisation and comparison of the activation of human prothrombin and human prethrombin-2 by the two ecarin preparations was made with a chromogenic assay at pH 8. Prothrombin and prethrombin-2, respectively, were cleaved by ecarin and the subsequent cleavage of the chromogenic thrombin substrate, H-CHG-Ala-Arg-pNa, by the activation products meizothrombin and thrombin, respectively, was followed by the change in A_405_ in time. The ecarin activity was calculated from the slope of the first derivative, dA_405_/dt versus time. The whole time curve was recorded at 0.1 s intervals, allowing calculation of the first derivative of the whole progress curve to estimate the conversion rate, *id est* the reaction rate of ecarin. For derivation the Savisky-Golay smoothing factor was used with 4th polynomial order and a window width of 9 data points.

The measurements were performed on Coatron M2 analyser (TECO Medical Instruments GmbH, Neufahrn, Germany). First 125 μl of the solution with different concentrations of prothrombin or prethrombin-2 was added to the microplate at room temperature, then 25 μl 6 mM H-CHG-Ala-Arg-pNa at 37 °C, and, after 1 min incubation in the analyser at 37 °C, 50 μl ecarin solution (0.1 EU/ml, to final 0.025 EU/ml). The reaction curves were recorded using Software TECMONI (TECO Medical Instruments GmbH). The raw data were exported and stored in an Excel file (Microsoft^®^) and transferred to GraFit version 5.0.13 (Erithacus, Software Limited) for determination of the first derivative and calculation of the rate. To ensure that the concentration of the chromogenic substrate was in excess during the whole reaction so that the rate of the chromogenic reaction depends on the prothrombin concentration alone, only A_405_ values below 0.3 were evaluated.

## Results and Discussion

### Activation of r-Ecarin

**Fig. 1:**
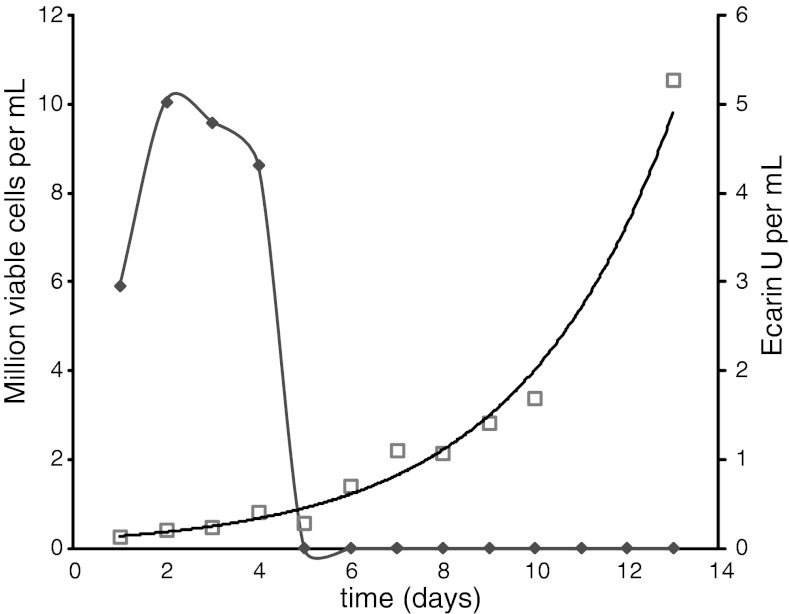
Activation of r-ecarin in cell culture. Cell culture samples were assayed for activity during the incubation time, as described in Methods with 1 mM S-2238 and 1 mg/L (14 nM) prothrombin, using native ecarin from Sigma as standard. Cell density million cells/ml (*diamonds*); Active Ecarin EU/ml (*squares*)

**Fig. 2:**
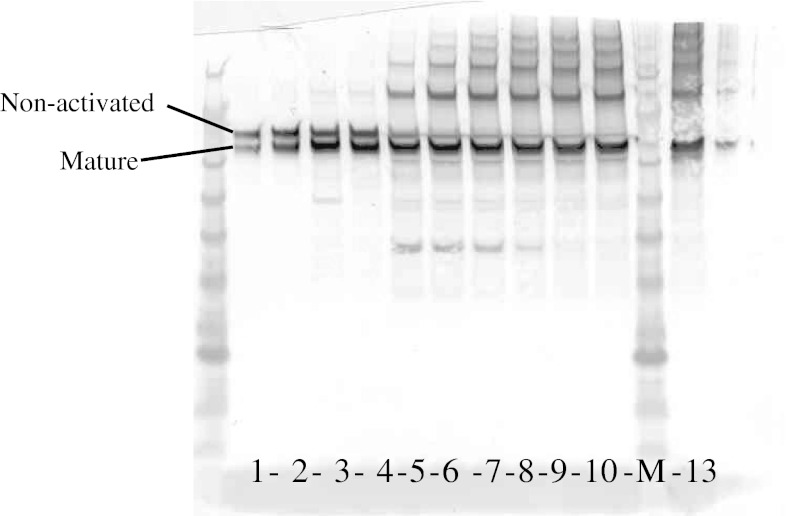
Western blot analyses of r-ecarin in cell culture samples. *Numbers* in the Figure denote day of sample collection from the cell culture, and *M* denotes molecular weight marker (See *Blue* +2 from Invitrogen) (Color figure online)

Ecarin is expressed as a pro-protein and removal of the pro-peptide is necessary for optimal ecarin activity. We found that removal of the pro-peptide was obtained by continued incubation of the cell culture for at least 7 days after the death of the ecarin-producing cells. After the death of the ecarin-producing cells the r-ecarin activity (Fig. 1) and the amount of mature r-ecarin increased (Fig. 2).

The activity of the r-ecarin in the culture supernatant from the clone 11B9b used for this work was calculated to be 3.1 EU/ml, both with the activity assay with H-CHG-Ala-Arg-pNa at pH 8 and the JenAffin ecarin standard and with the Sigma standard and the chromogenic thrombin substrate S-2238 at pH 7.4.

### Conversion of Prothrombin to Thrombin by r-Ecarin

To estimate the amount of ecarin-containing culture needed for converting prothrombin into thrombin, test digestions were performed. Diluted ecarin-containing culture supernatant samples were mixed with 1 g/l prothrombin in PBS buffer and incubated at 37 °C for 1–3 h. The digested samples were then analysed by SDS-PAGE and the amount of r-ecarin needed for complete conversion of prothrombin into thrombin was estimated. With this information available we calculated that one litre of ecarin-containing culture supernatant containing 7000 EU could convert 64 g of prothrombin into thrombin in less than 3 h at 37 °C.

### Enzyme Kinetic Properties of Native and r-Ecarin

**Fig. 3:**
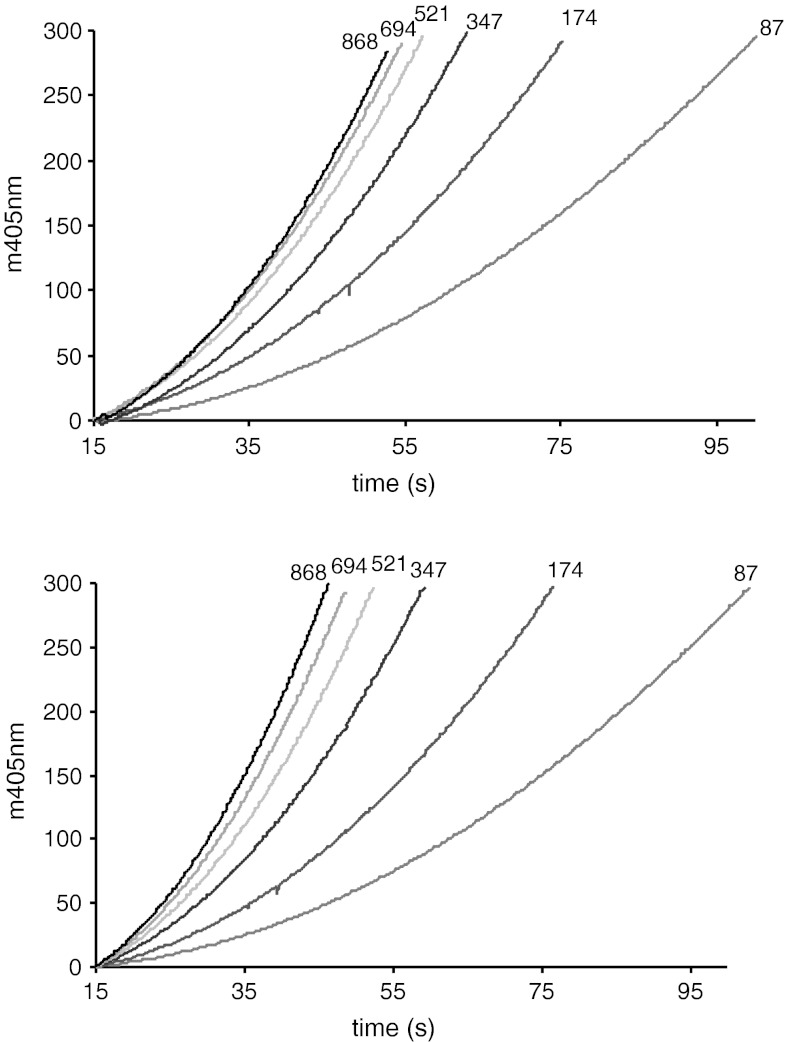
Cleavage of human prothrombin by ecarin. Ecarin (final 0.025 EU/ml) was added to prothrombin at different concentrations (final nM conc. as indicated) in Tris buffer at pH 8 at 37 °C with 0.75 mM H-CHG-Ala-Arg-pNa, monitored at 405 nm: A_405_ is plotted versus time for the different prothrombin concentrations. *Upper plot* native ecarin, *lower plot* r-ecarin

For both native and r-ecarin, the reaction rate of prothrombin cleavage was dependent on the prothrombin concentration and increased rapidly in the concentration range between 87 and 694 nM prothrombin although it did not approach a maximal rate at the highest prothrombin concentration, 868 mM, used (Fig. 3).

**Fig. 4:**
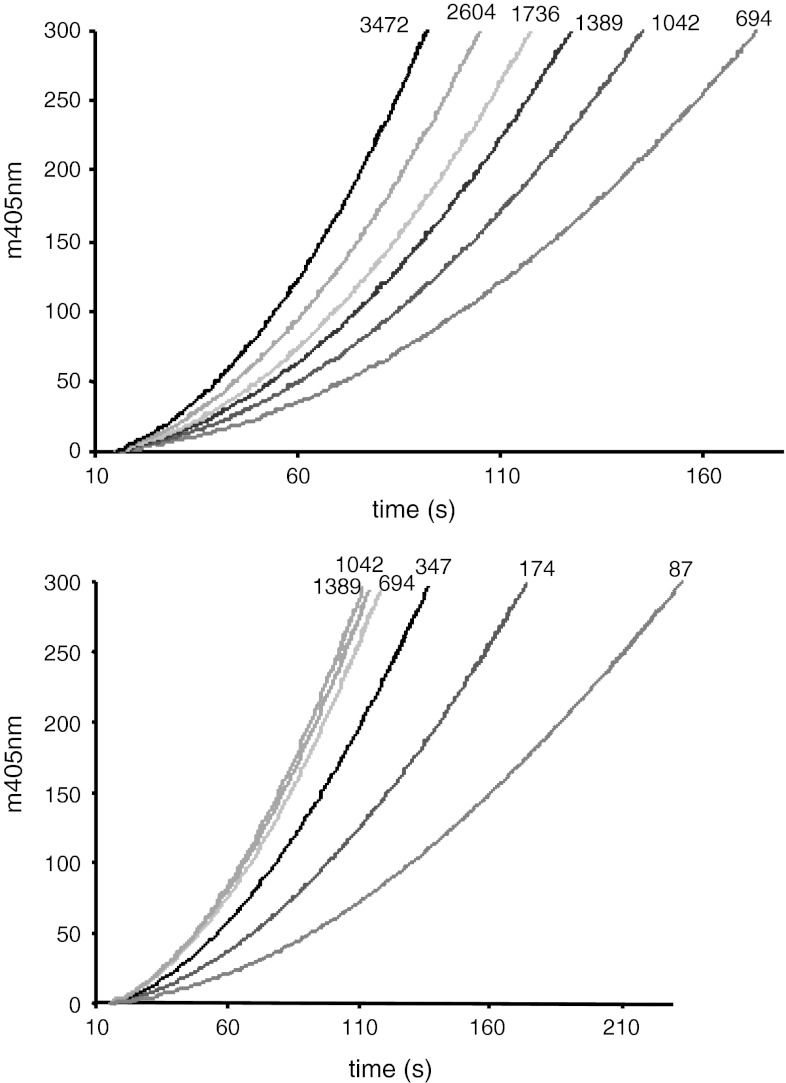
Cleavage of human prethrombin-2 by ecarin. Ecarin (final 0.025 EU/ml) was added to prethrombin-2 at different concentrations (final nM conc. as indicated) in Tris buffer at pH 8 at 37 °C with 0.75 mM H-CHG-Ala-Arg-pNa, monitored at 405 nm: A_405_ is plotted versus time for the different prethrombin-2 concentrations. *Upper plot* native ecarin, *lower plot* r-ecarin

In Fig. 4 the curves for the conversion of prethrombin-2 both by r-ecarin and by native ecarin are shown. The ecarin cleavage of prethrombin-2 was much slower than found for prothrombin (Fig. 3). In contrast to for prothrombin, the reaction curves for the cleavage of prethrombin-2 by native ecarin and the following cleavage of the chromogenic substrate are not comparable to the reaction curves of prethrombin-2 cleavage by r-ecarin. Only for r-ecarin, the reaction rate of prethrombin-2 cleavage increased in a concentration range between 87 and 347 nM prethrombin-2 and approached a maximal rate at the highest prethrombin-2 concentrations. With native ecarin the reaction rate of prethrombin-2 cleavage slowly increased over the whole concentration range tested, without reaching a plateau.

**Fig. 5:**
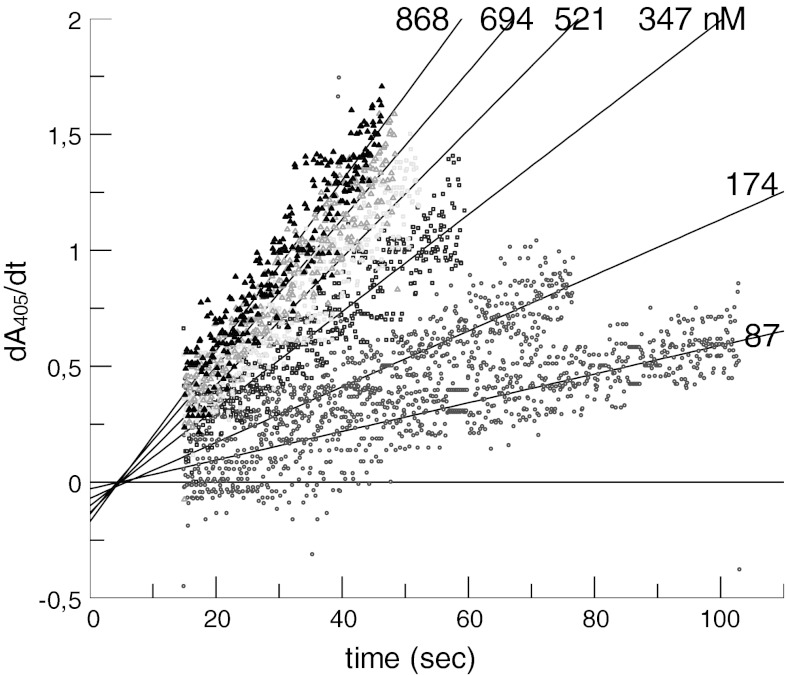
Activation of prothrombin by r-ecarin from thrombin activity. The recarin curves in Fig. 3 were analysed by calculation of the first derivative; dA_405_/dt plotted versus time (first derivative using 9 data points window width and 4th polynomial order) with final nM conc. prothrombin as indicated

**Fig. 6:**
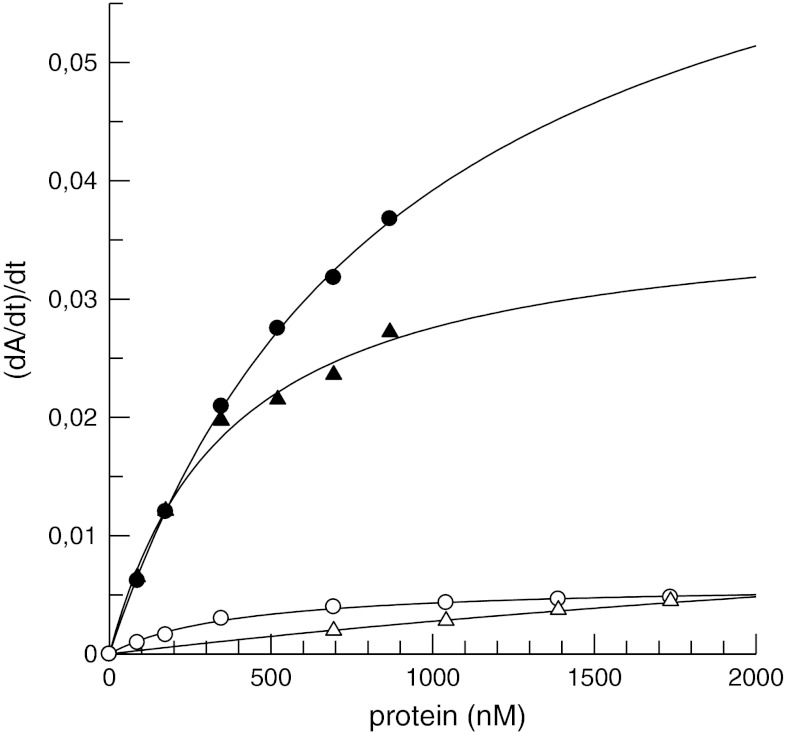
Estimation of *K*_m_ and V_max_ for pre- and prothrombin with ecarin. The slopes of the curves as exemplified in Fig. 5 are plotted versus the protein concentration to obtain the data for prothrombin (*closed symbols*) with r-ecarin (*circles*) or native ecarin (*triangles*). Similarly, the data for the other curves were deduced from Fig. 3 and 4, but with open symbols for prethrombin-2 as the protein substrate. The *lines* were drawn by non-linear regression applying the Michaelis–Menten equation

For the more accurate comparison of the reaction rates, the curves as shown in Fig. 3 and 4 were evaluated by the determination of the first derivative, dA/dt, see example for r-ecarin with prothrombin in Fig. 5. By linear regression the slopes for each prothrombin concentration were then calculated, representing the ecarin rate, and plotted versus the concentration of prothrombin and prethrombin-2, respectively, see Fig. 6. The kinetic constants were estimated by non-linear curve fitting to the simple Michaelis–Menten equation. The only complete set of data points, allowing a reliable estimate of the kinetic constants were thus obtained with prethrombin-2 and r-ecarin, with a defined *K*_m_ of 0.4 μM. For the three other curves the *K*_m_ values were less well defined, because of restrictions of the data sampling. For prothrombin the *K*_m_ value were roughly estimated to be about 0.9 μM and 0.4 μM with recombinant respectively native ecarin. The cleavage of prothrombin both by recombinant and native ecarin was comparable at prothrombin concentrations below 250 nM, see Fig. 6. However, at higher prothrombin concentrations far higher activity was found with r-ecarin than with native ecarin since the rate of hydrolysis with r-ecarin continued to increase, although apparently with native ecarin it levelled off.

### Discussion

We have successfully produced recombinant ecarin with high yield in CHO cells and with an activity comparable to the ecarin isolated from snake venom.

Although we successfully expressed mature ecarin in *E*. *coli*, this protein was obtained as inclusion bodies despite the presence of a secretory leader sequence and successful processing of this leader sequence (results not shown). The unusual amino acid composition of the mature ecarin containing 35 cysteine residues may be an explanation for the poor solubility and potentially also to the extreme stability of r-ecarin in the presence of dead CHO cells; the r-ecarin was found to be stable for months at room temperature with debris of the host cells present (results not shown). The larger molecular weight of the mature ecarin produced in CHO (~70 kDa) compared to the *E*. *coli* produced ecarin (~50 kDa), suggests that the CHO-produced ecarin is glycosylated (data not shown). Nishida et al. [6] predicted that there are 4–5 glycosylation sites in the mature ecarin. Glycosylation may be important to the solubility of ecarin, further explaining the inclusion bodies obtained in *E*. *coli*.

During purification experiments with the r-ecarin we noted that the mature ecarin is prone to aggregation already at low concentrations. In snake venom native ecarin may interact with other proteins that prevent aggregation and reduce stability; solutions containing native ecarin are not stable at room temperature. Western blot analyses of r-ecarin in culture samples suggest that aggregates accumulate simultaneously with the processing to mature ecarin (Fig. 2).

The activity of r-ecarin and native ecarin was comparable at prothrombin concentrations up to 250 nM. At higher prothrombin concentrations the activity for r-ecarin was higher than for native ecarin with an approximate double turnover. This finding is consistent with the rapid conversion by r-ecarin of prothrombin into thrombin. The apparent different affinities of native and r-ecarin for prethrombin-2 suggest that different proteases could be present in the native ecarin preparation, since the kinetics with r-ecarin was better defined. The reason for the difference between native and r-ecarin in turnover at high prothrombin concentrations is not understood, but it can be speculated that proteins interacting with native ecarin in snake venom affect the catalytic properties. Native ecarin preparations available to us were not sufficiently pure to exclude this possibility.

Ecarin purified from snake venom is currently used as prothrombin activator in diagnostic reagents for measurement of total prothrombin in plasma or for quantitative determination of direct thrombin inhibitors [7]. The use of components purified from snake venoms for such purposes has many drawbacks. Many of the snake species are endangered and the snakes have to be bred in special facilities. Handling of snakes and the venom is potentially dangerous as in addition to the prothrombin activators many other toxic components are present. Furthermore, differences among snake populations and in purification procedures create batch to batch variability. Development of recombinant ecarin will offer a more standardised activator of prothrombin.

In conclusion, r-ecarin was identified as a promising candidate for replacement of native ecarin in assays utilizing conversion of prothrombin to thrombin.

## Abbreviations

Glx: γ-Carboxyglutamate
r-: Recombinant
CHO: Chinese Hamster Ovary
